# 1-Hydroxymethyl-3,12-dioxa-14-aza­tetracyclo[9.2.1.0^4,14^.0^5,10^]tetradecane

**DOI:** 10.1107/S1600536808007976

**Published:** 2008-03-29

**Authors:** Xi-Shi Tai, Yi-Min Feng, Fan-Yuan Kong

**Affiliations:** a Department of Chemistry and Chemical Engineering, Weifang University, Weifang 261061, People’s Republic of China

## Abstract

In the title fused-ring compound, C_12_H_13_NO_3_, the two five-membered C_3_NO rings both approximate to envelope conformations with C atoms in the flap positions. The OH group of the pendant CH_2_OH unit is disordered over two positions in a 0.528 (5):0.472 (5) ratio. One of the OH groups participates in an O—H⋯N hydrogen bond, generating centrosymmetric dimers in the crystal structure.

## Related literature

For related literature, see: Tai *et al.* (2003 ▶).
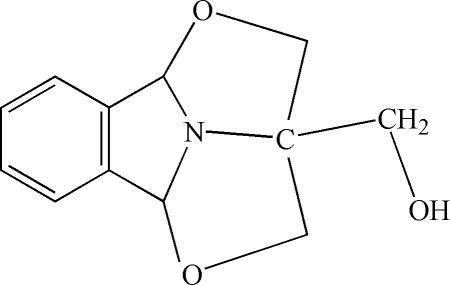


## Experimental

### 

#### Crystal data

C_12_H_13_NO_3_
*M* *_r_* = 219.23
Monoclinic, 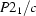

*a* = 6.5045 (9) Å
*b* = 7.1799 (10) Å
*c* = 22.394 (2) Å
β = 94.516 (2)°
*V* = 1042.6 (2) Å^3^
*Z* = 4
Mo *K*α radiation
μ = 0.10 mm^−1^
*T* = 298 (2) K
0.40 × 0.21 × 0.12 mm

#### Data collection

Bruker SMART CCD diffractometer
Absorption correction: multi-scan (*SADABS*; Bruker, 2000 ▶) *T*_min_ = 0.961, *T*_max_ = 0.988
5012 measured reflections
1936 independent reflections
1172 reflections with *I* > 2σ(*I*)
*R*_int_ = 0.072

#### Refinement

*R*[*F* ^2^ > 2σ(*F* ^2^)] = 0.098
*wR*(*F* ^2^) = 0.287
*S* = 1.03
1936 reflections
150 parameters
H-atom parameters constrained
Δρ_max_ = 0.39 e Å^−3^
Δρ_min_ = −0.30 e Å^−3^

### 

Data collection: *SMART* (Bruker, 2000 ▶); cell refinement: *SAINT* (Bruker, 2000 ▶); data reduction: *SAINT*; program(s) used to solve structure: *SHELXS97* (Sheldrick, 2008 ▶); program(s) used to refine structure: *SHELXL97* (Sheldrick, 2008 ▶); molecular graphics: *SHELXTL* (Sheldrick, 2008 ▶); software used to prepare material for publication: *SHELXTL*.

## Supplementary Material

Crystal structure: contains datablocks global, I. DOI: 10.1107/S1600536808007976/hb2709sup1.cif

Structure factors: contains datablocks I. DOI: 10.1107/S1600536808007976/hb2709Isup2.hkl

Additional supplementary materials:  crystallographic information; 3D view; checkCIF report

## Figures and Tables

**Table 1:** Hydrogen-bond geometry (Å, °)

*D*—H⋯*A*	*D*—H	H⋯*A*	*D*⋯*A*	*D*—H⋯*A*
O3—H3⋯N1^i^	0.82	2.11	2.882 (7)	156

Symmetry code: (i) 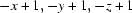.

**Fig. 1.**
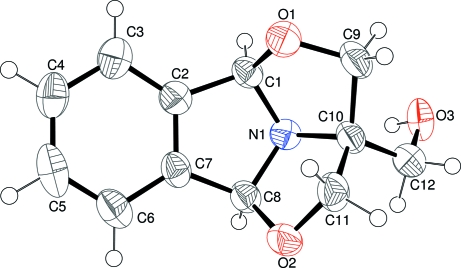
The molecular structure of (I) showing 50% displacement ellipsoids for the non-hydrogen atoms. Only one orientation of the disordered -CH2OH group is shown.

## supplementary crystallographic information

### Comment

As part of our ongoing studies of fused-ring systems (Tai *et al.*, 2003) we now report the synthesis and structure of the title compound, (I).

The C1/C2/C7/C8/N1 ring is almost planar (r.m.s. deviation from the mean plane = 0.004Å).  The C1/O1/C9/C10/N1 ring is a twisted envelope with C9 in the flap position. The C8/O2/C11/C10/N1 ring is a well defined envelope, with C11 deviating by 0.504 (7)Å from the mean plane of the other four atoms. The molecule of (I) is chiral, with C1 and C8 having R and S configurations respectively, in the arbitrarily chosen asymmetric molecule, but crystal symmetry generates a racemic mixture.

The pendant -CH_2_OH group is disordered over two orientations in almost equal proportions.  One of the orientations participates in an intermolecular O-H···N hydrogen bond (Table 1), leading to inversion dimers in the crystal.

### Experimental

Ortho-phthaladehyde (5 mmol) was added to a solution of trihydroxymethyl aminomethane (5 mmol) in 10 ml of ethanol.  The mixture was continuously stirred for 2 h at refluxing temperature, evaporating some ethanol, then, upon cooling, the solid product was collected by filtration and dried in vacuo (yield 58%). Colourless blocks of (I) were obtained by evaporation from a methanol solution after 10 days.

### Refinement

The H atoms were placed geometrically (C—H = 0.93–0.96 Å, O—H = 0.82 Å) and refined as riding with *U*_iso_(H) = 1.2*U*_eq_(C) or 1.5U_eq_(O).

### Crystal data

**Table:**

C_12_H_13_NO_3_	*F*_000_ = 464
*M**_r_* = 219.23	*D*_x_ = 1.397 Mg m^−^^3^
Monoclinic, *P*2_1_/*c*	Mo *K*α radiation λ = 0.71073 Å
Hall symbol: -P 2ybc	Cell parameters from 1373 reflections
*a* = 6.5045 (9) Å	θ = 3.0–23.3º
*b* = 7.1799 (10) Å	µ = 0.10 mm^−^^1^
*c* = 22.394 (2) Å	*T* = 298 (2) K
β = 94.516 (2)º	Block, colourless
*V* = 1042.6 (2) Å^3^	0.40 × 0.21 × 0.12 mm
*Z* = 4	

### Data collection

**Table:**

Bruker SMART CCD diffractometer	1936 independent reflections
Radiation source: fine-focus sealed tube	1172 reflections with *I* > 2σ(*I*)
Monochromator: graphite	*R*_int_ = 0.073
*T* = 298(2) K	θ_max_ = 26.0º
ω scans	θ_min_ = 1.8º
Absorption correction: multi-scan(SADABS; Bruker, 2000)	*h* = −8→7
*T*_min_ = 0.961, *T*_max_ = 0.988	*k* = −7→8
5012 measured reflections	*l* = −27→21

### Refinement

**Table:**

Refinement on *F*^2^	Hydrogen site location: inferred from neighbouring sites
Least-squares matrix: full	H-atom parameters constrained
*R*[*F*^2^ > 2σ(*F*^2^)] = 0.098	*w* = 1/[σ^2^(*F*_o_^2^) + (0.1727*P*)^2^ + 0.3664*P*] where *P* = (*F*_o_^2^ + 2*F*_c_^2^)/3
*wR*(*F*^2^) = 0.287	(Δ/σ)_max_ < 0.001
*S* = 1.03	Δρ_max_ = 0.39 e Å^−^^3^
1936 reflections	Δρ_min_ = −0.30 e Å^−^^3^
150 parameters	Extinction correction: SHELXL, Fc^*^=kFc[1+0.001xFc^2^λ^3^/sin(2θ)]^-1/4^
Primary atom site location: structure-invariant direct methods	Extinction coefficient: 0.079 (19)
Secondary atom site location: difference Fourier map	

### Special details

**Table:**

Geometry. All e.s.d.'s (except the e.s.d. in the dihedral angle between two l.s. planes) are estimated using the full covariance matrix. The cell e.s.d.'s are taken into account individually in the estimation of e.s.d.'s in distances, angles and torsion angles; correlations between e.s.d.'s in cell parameters are only used when they are defined by crystal symmetry. An approximate (isotropic) treatment of cell e.s.d.'s is used for estimating e.s.d.'s involving l.s. planes.
Refinement. Refinement of *F*^2^ against ALL reflections. The weighted *R*-factor *wR* and goodness of fit *S* are based on *F*^2^, conventional *R*-factors *R* are based on *F*, with *F* set to zero for negative *F*^2^. The threshold expression of *F*^2^ > σ(*F*^2^) is used only for calculating *R*-factors(gt) *etc*. and is not relevant to the choice of reflections for refinement. *R*-factors based on *F*^2^ are statistically about twice as large as those based on *F*, and *R*- factors based on ALL data will be even larger.

### Fractional atomic coordinates and isotropic or equivalent isotropic displacement parameters (Å^2^)

**Table:**

	*x*	*y*	*z*	*U*_iso_*/*U*_eq_	Occ. (<1)
N1	0.3226 (5)	0.5287 (5)	0.42690 (14)	0.0367 (9)	
O1	0.4295 (5)	0.6752 (5)	0.34186 (13)	0.0486 (10)	
O2	−0.0354 (5)	0.5594 (5)	0.42167 (15)	0.0511 (10)	
O3	0.4817 (11)	0.7928 (9)	0.5164 (3)	0.0524 (13)	0.528 (5)
H3	0.5091	0.6856	0.5269	0.079*	0.528 (5)
O3'	0.2177 (12)	0.9609 (10)	0.5081 (3)	0.0524 (13)	0.472 (5)
H3'	0.2718	1.0454	0.4903	0.079*	0.472 (5)
C1	0.4333 (7)	0.5026 (6)	0.37325 (18)	0.0378 (11)	
H1	0.5753	0.4617	0.3837	0.045*	
C2	0.3133 (7)	0.3565 (7)	0.33750 (19)	0.0403 (11)	
C3	0.3589 (8)	0.2725 (7)	0.2844 (2)	0.0494 (13)	
H3A	0.4819	0.2969	0.2673	0.059*	
C4	0.2158 (9)	0.1520 (7)	0.2581 (2)	0.0547 (14)	
H4	0.2433	0.0914	0.2229	0.066*	
C5	0.0313 (10)	0.1188 (8)	0.2829 (3)	0.0650 (16)	
H5	−0.0665	0.0418	0.2629	0.078*	
C6	−0.0101 (8)	0.1980 (7)	0.3367 (2)	0.0523 (13)	
H6	−0.1303	0.1699	0.3547	0.063*	
C7	0.1354 (7)	0.3223 (6)	0.36316 (19)	0.0409 (11)	
C8	0.1283 (7)	0.4262 (6)	0.42126 (19)	0.0402 (11)	
H8	0.1217	0.3386	0.4546	0.048*	
C9	0.4143 (8)	0.8132 (7)	0.3856 (2)	0.0495 (13)	
H9A	0.3567	0.9270	0.3679	0.059*	
H9B	0.5484	0.8405	0.4057	0.059*	
C10	0.2694 (7)	0.7288 (6)	0.42937 (19)	0.0399 (11)	
C11	0.0471 (7)	0.7326 (7)	0.4034 (2)	0.0474 (12)	
H11A	−0.0266	0.8366	0.4193	0.057*	
H11B	0.0395	0.7421	0.3601	0.057*	
C12	0.2945 (8)	0.8000 (9)	0.4924 (2)	0.0586 (15)	
H12A	0.2476	0.9282	0.4928	0.070*	0.528 (5)
H12B	0.2068	0.7277	0.5167	0.070*	0.528 (5)
H12C	0.2323	0.7085	0.5173	0.070*	0.472 (5)
H12D	0.4410	0.8004	0.5045	0.070*	0.472 (5)

### Atomic displacement parameters (Å^2^)

**Table:**

	*U*^11^	*U*^22^	*U*^33^	*U*^12^	*U*^13^	*U*^23^
N1	0.037 (2)	0.035 (2)	0.0380 (19)	0.0013 (16)	0.0021 (14)	0.0041 (15)
O1	0.067 (2)	0.0364 (19)	0.0437 (18)	−0.0037 (16)	0.0156 (15)	0.0067 (13)
O2	0.0313 (17)	0.047 (2)	0.076 (2)	−0.0037 (14)	0.0062 (14)	−0.0088 (16)
O3	0.065 (3)	0.031 (3)	0.059 (3)	0.001 (2)	−0.008 (2)	−0.008 (2)
O3'	0.065 (3)	0.031 (3)	0.059 (3)	0.001 (2)	−0.008 (2)	−0.008 (2)
C1	0.038 (2)	0.031 (2)	0.043 (2)	−0.0015 (18)	0.0019 (17)	0.0038 (18)
C2	0.040 (2)	0.037 (2)	0.044 (2)	0.0015 (19)	0.0009 (18)	0.0023 (19)
C3	0.059 (3)	0.043 (3)	0.045 (3)	0.009 (2)	−0.001 (2)	0.000 (2)
C4	0.066 (4)	0.038 (3)	0.058 (3)	0.010 (3)	−0.003 (2)	−0.010 (2)
C5	0.080 (4)	0.041 (3)	0.070 (4)	−0.006 (3)	−0.023 (3)	−0.012 (3)
C6	0.048 (3)	0.039 (3)	0.069 (3)	−0.007 (2)	−0.004 (2)	0.000 (2)
C7	0.042 (3)	0.029 (2)	0.050 (3)	0.0015 (19)	−0.0026 (19)	−0.0014 (18)
C8	0.041 (2)	0.034 (3)	0.045 (2)	−0.0039 (19)	0.0032 (18)	0.0006 (18)
C9	0.053 (3)	0.032 (3)	0.064 (3)	−0.011 (2)	0.006 (2)	0.000 (2)
C10	0.036 (2)	0.030 (2)	0.053 (3)	−0.0043 (18)	0.0030 (18)	−0.0027 (19)
C11	0.045 (3)	0.033 (3)	0.063 (3)	0.004 (2)	0.000 (2)	−0.006 (2)
C12	0.046 (3)	0.057 (4)	0.074 (3)	−0.014 (2)	0.012 (2)	−0.020 (3)

### Geometric parameters (Å, °)

**Table:**

N1—C8	1.460 (6)	C4—H4	0.9300
N1—C1	1.461 (6)	C5—C6	1.379 (8)
N1—C10	1.480 (6)	C5—H5	0.9300
O1—C9	1.402 (6)	C6—C7	1.398 (7)
O1—C1	1.424 (5)	C6—H6	0.9300
O2—C11	1.427 (6)	C7—C8	1.504 (6)
O2—C8	1.431 (5)	C8—H8	0.9800
O3—C12	1.293 (8)	C9—C10	1.537 (7)
O3—H3	0.8200	C9—H9A	0.9700
O3—H12D	0.3646	C9—H9B	0.9700
O3'—C12	1.317 (10)	C10—C12	1.498 (7)
O3'—H3'	0.8200	C10—C11	1.516 (6)
C1—C2	1.501 (6)	C11—H11A	0.9700
C1—H1	0.9800	C11—H11B	0.9700
C2—C7	1.355 (7)	C12—H12A	0.9700
C2—C3	1.386 (7)	C12—H12B	0.9700
C3—C4	1.370 (8)	C12—H12C	0.9700
C3—H3A	0.9300	C12—H12D	0.9700
C4—C5	1.382 (9)		
			
C8—N1—C1	110.1 (3)	C7—C8—H8	110.3
C8—N1—C10	106.8 (3)	O1—C9—C10	104.3 (4)
C1—N1—C10	106.7 (3)	O1—C9—H9A	110.9
C9—O1—C1	105.7 (3)	C10—C9—H9A	110.9
C11—O2—C8	106.4 (3)	O1—C9—H9B	110.9
C12—O3—H3	109.5	C10—C9—H9B	110.9
H3—O3—H12D	118.6	H9A—C9—H9B	108.9
C12—O3'—H3'	109.5	N1—C10—C12	111.0 (4)
O1—C1—N1	107.7 (3)	N1—C10—C11	102.8 (3)
O1—C1—C2	110.9 (3)	C12—C10—C11	112.7 (4)
N1—C1—C2	105.0 (4)	N1—C10—C9	101.7 (4)
O1—C1—H1	111.0	C12—C10—C9	116.1 (4)
N1—C1—H1	111.0	C11—C10—C9	111.2 (4)
C2—C1—H1	111.0	O2—C11—C10	104.1 (4)
C7—C2—C3	122.2 (5)	O2—C11—H11A	110.9
C7—C2—C1	109.1 (4)	C10—C11—H11A	110.9
C3—C2—C1	128.6 (4)	O2—C11—H11B	110.9
C4—C3—C2	117.4 (5)	C10—C11—H11B	110.9
C4—C3—H3A	121.3	H11A—C11—H11B	109.0
C2—C3—H3A	121.3	O3—C12—O3'	106.8 (5)
C3—C4—C5	121.2 (5)	O3—C12—C10	114.0 (5)
C3—C4—H4	119.4	O3'—C12—C10	122.3 (6)
C5—C4—H4	119.4	O3—C12—H12A	108.7
C6—C5—C4	121.0 (5)	C10—C12—H12A	108.7
C6—C5—H5	119.5	O3—C12—H12B	108.7
C4—C5—H5	119.5	C10—C12—H12B	108.7
C5—C6—C7	117.5 (5)	H12A—C12—H12B	107.6
C5—C6—H6	121.2	O3—C12—H12C	99.0
C7—C6—H6	121.2	O3'—C12—H12C	104.9
C2—C7—C6	120.5 (4)	C10—C12—H12C	106.9
C2—C7—C8	111.3 (4)	H12A—C12—H12C	119.4
C6—C7—C8	128.1 (4)	O3'—C12—H12D	107.9
O2—C8—N1	107.6 (4)	C10—C12—H12D	107.2
O2—C8—C7	114.2 (3)	H12A—C12—H12D	107.3
N1—C8—C7	103.9 (3)	H12B—C12—H12D	116.9
O2—C8—H8	110.3	H12C—C12—H12D	106.7
N1—C8—H8	110.3		
			
C9—O1—C1—N1	27.5 (4)	C10—N1—C8—C7	120.2 (4)
C9—O1—C1—C2	141.9 (4)	C2—C7—C8—O2	117.6 (4)
C8—N1—C1—O1	110.3 (4)	C6—C7—C8—O2	−65.0 (6)
C10—N1—C1—O1	−5.3 (4)	C2—C7—C8—N1	0.7 (5)
C8—N1—C1—C2	−8.0 (5)	C6—C7—C8—N1	178.1 (5)
C10—N1—C1—C2	−123.5 (4)	C1—O1—C9—C10	−37.8 (5)
O1—C1—C2—C7	−107.7 (4)	C8—N1—C10—C12	101.6 (4)
N1—C1—C2—C7	8.4 (5)	C1—N1—C10—C12	−140.7 (4)
O1—C1—C2—C3	68.2 (6)	C8—N1—C10—C11	−19.1 (4)
N1—C1—C2—C3	−175.7 (4)	C1—N1—C10—C11	98.6 (4)
C7—C2—C3—C4	0.2 (7)	C8—N1—C10—C9	−134.3 (3)
C1—C2—C3—C4	−175.2 (5)	C1—N1—C10—C9	−16.6 (4)
C2—C3—C4—C5	1.3 (8)	O1—C9—C10—N1	33.4 (4)
C3—C4—C5—C6	−3.6 (9)	O1—C9—C10—C12	154.0 (4)
C4—C5—C6—C7	4.1 (8)	O1—C9—C10—C11	−75.4 (5)
C3—C2—C7—C6	0.4 (7)	C8—O2—C11—C10	−34.6 (4)
C1—C2—C7—C6	176.7 (4)	N1—C10—C11—O2	32.8 (4)
C3—C2—C7—C8	178.1 (4)	C12—C10—C11—O2	−86.7 (5)
C1—C2—C7—C8	−5.7 (5)	C9—C10—C11—O2	140.9 (4)
C5—C6—C7—C2	−2.5 (7)	N1—C10—C12—O3	63.2 (6)
C5—C6—C7—C8	−179.8 (5)	C11—C10—C12—O3	177.9 (5)
C11—O2—C8—N1	22.8 (4)	C9—C10—C12—O3	−52.2 (7)
C11—O2—C8—C7	−92.0 (4)	N1—C10—C12—O3'	−165.7 (6)
C1—N1—C8—O2	−116.7 (4)	C11—C10—C12—O3'	−51.1 (7)
C10—N1—C8—O2	−1.2 (4)	C9—C10—C12—O3'	78.8 (7)
C1—N1—C8—C7	4.8 (4)		

### Hydrogen-bond geometry (Å, °)

**Table:**

*D*—H···*A*	*D*—H	H···*A*	*D*···*A*	*D*—H···*A*
O3—H3···N1^i^	0.82	2.11	2.882 (7)	156

Symmetry codes: (i) −*x*+1, −*y*+1, −*z*+1.
